# Loss of Type 1 Pili and Flagella in Uropathogenic *Escherichia coli* Leads to Reduced Phagocytosis by Human and Murine Monocytes

**DOI:** 10.3390/pathogens14100968

**Published:** 2025-09-25

**Authors:** William R. Schwan

**Affiliations:** Department of Microbiology, University of Wisconsin-La Crosse, La Crosse, WI 54601, USA; wschwan@uwlax.edu

**Keywords:** type 1 fimbriae, type 1 pili, flagella, uropathogenic *Escherichia coli*

## Abstract

Background: Uropathogenic *Escherichia coli* (UPEC) is the number one cause of urinary tract infections (UTIs) in humans. The ability to bind to uroepithelial cells through type 1 pili and ascend the urinary tract via flagella is important in the early stages of a UTI. However, both type 1 pili and flagella can also target the bacteria for elimination via monocytes/macrophages later in a UTI. We hypothesized that the loss of both type 1 pili and flagella on the UPEC cells would make them less likely to be phagocytized by phagocytic cells. Methods: In this study, Δ*fimA*, Δ*fliC*, and Δ*fimA* Δ*fliC* mutants were compared to the wild type UPEC strain NU149 in phagocytosis assays using human and murine monocytic cell lines. Results: A Δ*fimA* Δ*fliC* double mutant was phagocytized significantly less than the wild type strain. Conclusion: The data show that the loss of both type 1 pili and flagella expression on the UPEC cells reduces phagocytosis of the bacteria by human and murine monocytes. Although type 1 pili and flagella are important for establishing a UTI and ascension into the kidneys, the loss of these proteinaceous structures may allow the UPEC cells to evade the innate immune defenses in certain environments within the human body.

## 1. Introduction

Worldwide, approximately 405 million urinary tract infections (UTIs) occur each year [1]. Within the United States, around 10.5 million women contract UTIs each year [2], with 75% of uncomplicated UTIs caused by uropathogenic *Escherichia coli* (UPEC) [3]. Over 100,000 people are hospitalized each year due to UPEC isolates, some because their pyelonephritis leads to sepsis [4].

UPEC isolates establish UTIs by first adhering to mannose laden urethral epithelial cells via type 1 pili [5,6]. The type 1 pili are long, thin filamentous appendages that are predominantly composed of hundreds of FimA monomers encoded by the *fimA* gene [7,8]. At the tip of the type 1 pilus is the FimH adhesin that binds to mannosylated moieties [9,10,11], including those found on urethral and bladder epithelial cells [5,6,12,13,14]. Besides having a role in the adherence to uroepithelial cells, type 1 pili are also tied to the invasion of these host cells where they form intracellular pod-like bacterial communities (IBCs) [15,16]. Most UPEC strains are able to produce type 1 pili [17,18].

After creating a beachhead in the urethra via type 1 pili, the UPEC cells can first ascend to the bladder and sometimes climb up into the kidneys because of the action of flagella. Flagella confer motility to UPEC cells, and more than 30 different proteins are tied to motility and chemotaxis in *E. coli* [19]. An *E. coli* flagellum is separated into the basal body, the hook, and the filament [20]. The flagellar filament is primarily composed of tens of thousands of FliC flagellin subunits [19]. Most UPEC strains produce flagella [21]. The loss of flagella on the UPEC cells hinders the ascension of the bacteria into the kidneys of the host [22,23,24,25]. Thus, flagella are needed for the bacteria to move up the urinary tract in the early stages of a UTI.

Although type 1 pili and flagella are critical in the early stages of a urinary tract infection, do the UPEC cells still need these highly immunogenic surface structures as the urinary tract infection progresses? Over time, UPEC cells show a decline in type 1 pili expression in the murine and human urinary tracts [26,27,28]. Within murine kidneys, the loss of type 1 piliated UPEC cells is more pronounced after a 5–7-day time period post-infection compared to after the first day [27,28]. Moreover, the expression of flagella by UPEC cells is also reduced as time elapses within the urinary tract [23,29,30].

In this study, UPEC strain NU149 was used [28]. The strain was isolated from a woman that had cystitis and has been used to study UPEC pathogenesis [24,28]. Mutants of strain NU149 missing the *fimA* and/or the *fliC* gene were examined in a phagocytosis assay compared to the wild type strain to see if the loss of type 1 pili and/or flagella had an effect on their ability to be phagocytized by monocytes. We show that the strain with a deletion of both the *fimA* and *fliC* genes was phagocytized the least, suggesting there may be a beneficial role for the loss of both type 1 pili and flagella on UPEC cells in certain environments to avoid phagocytosis by murine and human monocytes.

## 2. Materials and Methods

### 2.1. Bacterial Strains, Plasmids, and Growth Media

All strains and plasmids used in the study are listed in Table 1. The UPEC strain NU149 [28] was grown in Luria broth (LB) as previously described [31]. Strain DH5α was used as a recipient for transformations. Luria agar (LA) was used during the transformations with the addition of the following antibiotics: kanamycin, 40 μg/mL or ampicillin, 100 μg/mL (Sigma Aldrich, St. Louis, MO, USA). All media used in the study were purchased from Thermo Fisher Scientific, Pittsburgh, PA, USA. The λ Red recombinase system with plasmids pKD4, pDD46, and pCP20 was used as previously described [32].

### 2.2. Construction of the ΔfimA Single Mutant and ΔfimA ΔfliC Double Mutant

To create the Δ*fimA* and Δ*fimA* Δ*fliC* mutants in strain NU149, the λ Red recombinase system was used [32]. For both mutants, the primer pair FimA7 (5′ GACCGTTCACTTTAAAGGGGAAGTTGTTAACGCCGCTTGTGTGTAGGCT GGAGCTGCTTCG 3′) and FimA8 (5′ACCCGTTCTGTCCAGGATCTGCACACCAACGTTTGTTGCGCATATGAATATCCTCCTTAG 3′) was used to create the PCR product, using pKD4 plasmid DNA as a template. The PCR conditions that were used were an initial denaturation at 95 °C for 5 min, followed by 35 cycles of 95 °C, 1 min; 55 °C, 1 min; and 72 °C, 2 min. The resulting PCR product was processed as described previously [24]. Electroporations were performed with the PCR product on strains NU149/pKD46 to create the Δ*fimA* mutant or NU149 FliC2/pKD46 to create the Δ*fimA* Δ*fliC* double mutant, selecting for transformants on LA with kanamycin. One transformant for the Δ*fimA* mutation NU149 FimA1 and one transformant for the Δ*fimA* Δ*fliC* double mutation NU149 FimA1 FliC2 were chosen for further analyses. Removal of the kanamycin resistance gene in both strains was accomplished using pCP20 as previously noted [32]. Confirmation of the *fimA* deletion was performed using the FimA1 (5′ GAAGTTGTTAACGCCGCTTG 3′) and FimA2 (5′ GCACACCAACGTTTGAAGCG 3′) primer pair under the following conditions: initial denaturation at 95 °C for 5 min, followed by 30 cycles of 95 °C, 1 min; 57 °C, 1 min; and 72 °C, 1 min using a previous protocol [33].

### 2.3. Hemagglutination Assay (HA)

The HA assays were performed with 1% guinea pig erythrocytes (Hardy Diagnostics, Santa Maria, CA, USA) as previously described [34], with and without 1% mannose (*w*/*v*) added (Sigma Aldrich). The titers represent the average of three separate runs.

### 2.4. Motility Assay

A soft agar motility assay was performed as previously described [35]. Strains were inoculated into the center of the agar plate, and the amount of bacterial spread was measured after 6 h post-inoculation at 37 °C. A total of three assessments were conducted per strain, and the mean was calculated for each strain.

### 2.5. Transmission Electron Microscopy

Samples from all of the strains grown in LB overnight at 37 °C were attached to Formvar grids (Thermo Fisher Scientific, Pittsburgh, PA, USA) and processed as described by Hultgren and Hidvegi [36]. Grids were observed using a JEOL 1200EXII transmission electron microscope (JEOL USA, Inc. Peabody, MA, USA). Images were gathered at ×25,000 or ×50,000 magnification using a Gatan 791 digital camera running Digital Micrograph version 3.1 (Gatan, Inc., Pleasanton, CA, USA). A rank sharpening filter was applied globally to the images to help visualize the pili and flagella on the bacteria. Between 50 and 100 cells were examined for each strain.

### 2.6. Phagocytosis Assay

Murine monocyte cell line J774A.1 (American Type Culture Collection [ATCC], Manassas, VA, USA) and human monocytic cell line U937 (ATCC) were used for the assays. The tissue culture cells were diluted to 1 × 10^6^ per well, and phorbol 12-myristrate 13-acetate (PMA, Sigma Aldrich, St. Louis, MO, USA) was added to the wells at a concentration of 50 ng/mL for 24 h to differentiate the monocytes into macrophages. *E. coli* strains were grown overnight in 5 mL of LB grown statically at 37 °C to maximize type 1 pilus expression. Overnight cultures were diluted to 1 × 10^6^ CFU/mL in RPMI medium, and 1 mL aliquots were added to 1 × 10^6^ PMA treated J774A.1 or U937 monolayers in 24-well tissue culture plates in duplicate for each strain tested for a multiplicity of infection (MOI) of 1:1, which would align with the MOI you might find in vivo. All monolayers were incubated for 2 h at 37 °C. The tissue culture cells containing the bacteria were then scrapped out of each well and placed into plastic tubes. Each aliquot was 10-fold serially diluted in PBS and 100 uL aliquots plated in duplicate onto LA plates. The plates were allowed to incubate at 37 °C overnight, and the viable counts were determined for each *E. coli* strain. Each assay was repeated two times for each bacterial strain and monocyte line tested. A mean ± standard deviation was calculated for each bacterial strain and monocyte cell line used.

For determining the intracellular uptake of bacteria into the monocytes, the same procedure as described above was used with the following changes. After the bacteria were added to the monocytes, the monolayers with bacteria were incubated for 1 h at 37 °C. Gentamicin was then added at a concentration of 50 μg/mL and incubated at 37 °C for 1 h to kill the extracellular *E. coli* cells. Tissue culture cells were then rinsed three times with PBS, 1 mL aliquots of 0.1% Triton X-100 (Sigma Aldrich) were added to each well, and the tissue culture cells containing the bacteria were scrapped out of each well and placed into plastic tubes. Each aliquot was 10-fold serially diluted in PBS and 100 μL aliquots plated in duplicate onto LA plates. The plates were allowed to incubate at 37 °C overnight, and the viable counts were determined for each *E. coli* strain. Each assay was repeated two more times for each bacterial strain and monocyte line tested. A mean ± standard deviation was calculated for each bacterial strain and monocyte cell line used.

### 2.7. Statistics

A two-tailed paired Student’s *t* test was used for statistical analyses. *p* values ≤ 0.05 were considered significant.

## 3. Results

### 3.1. Confirming the Loss of Type 1 Pili and Flagella in the Strain NU149 Mutants

In this study, Δ*fimA* (NU149 FimA1) and Δ*fimA* Δ*fliC* double mutant strains (NU149 FimA1 FliC2; Table 1) were constructed and tested in both motility and hemagglutination assays compared to the wild type NU149 strain and a previously constructed Δ*fliC* mutant strain [24]. The wild type strain NU149 displayed a high hemagglutination titer (HA), as well as a high degree of motility on soft agar (Table 2). The addition of 1% mannose (*w*/*v*) reduced the HA titer down to 0 for the wild type strain, confirming the mannose sensitivity. The Δ*fimA* mutant showed an HA titer of 0, indicating a loss of type 1 pili, but the motility on soft agar was similar to the wild type strain. When the Δ*fliC* mutant was tested, the HA titer matched the wild type strain. However, the mutant strain’s motility fell to 5 mm, which was equal to a past study [24]. Lastly, the Δ*fimA* Δ*fliC* double mutant strain demonstrated an HA titer of 0 and a very low motility on soft agar, indicating the loss of both type 1 pili and flagella.

**Figure 1:**
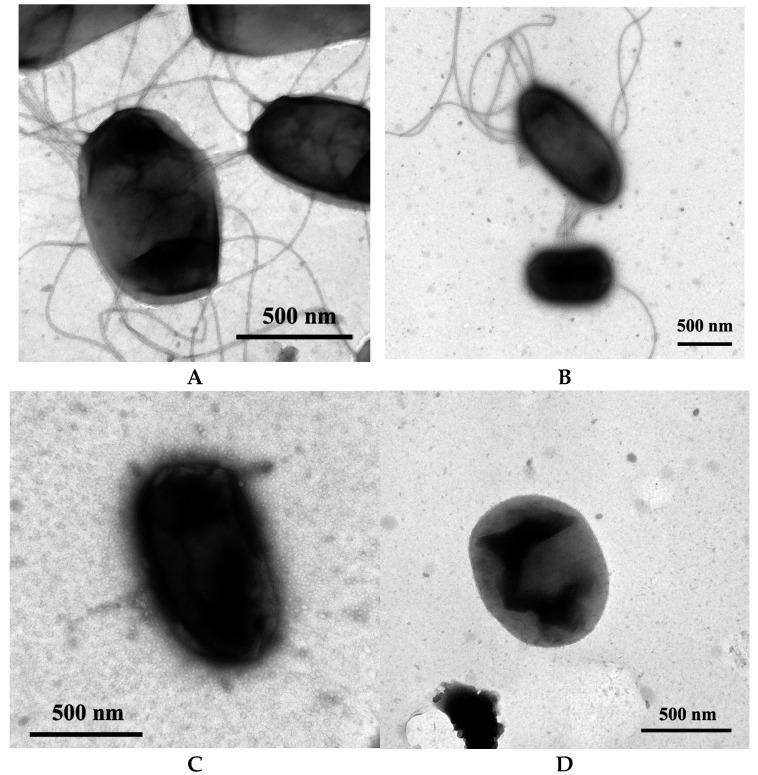
Electron micrographs of wild type NU149 and three mutant strains lacking type 1 pili and/or flagella. The panels are displayed as follows: (**A**) wild type, (**B**) Δ*fimA* mutant, (**C**) Δ*fliC* mutant, and (**D**) Δ*fimA* Δ*fliC* double mutant. A bar representing 500 nm has been added.

To confirm the NU149 FimA1, NU149 FliC2, and NU149 FimA1 FliC2 strains had lost the expression of type 1 pili and/or flagella, the strains were examined through transmission electron microscopy. The wild type strain NU149 expressed both type 1 pili and flagella (Figure 1A). An examination of the Δ*fimA* mutant showed flagella expression, but the loss of type 1 pili expression (Figure 1B). The Δ*fliC* mutant expressed type 1 pili; however, no flagella were observed (Figure 1C). Neither type 1 pili nor flagella were seen on the Δ*fimA* Δ*fliC* strain cells (Figure 1D). Thus, electron microscopy confirmed the loss of expression of these surface structures on the respective strains.

### 3.2. Fewer fimA fliC Double Mutant Cells Phagocytized by Human and Murine Monocytes

**Figure 2:**
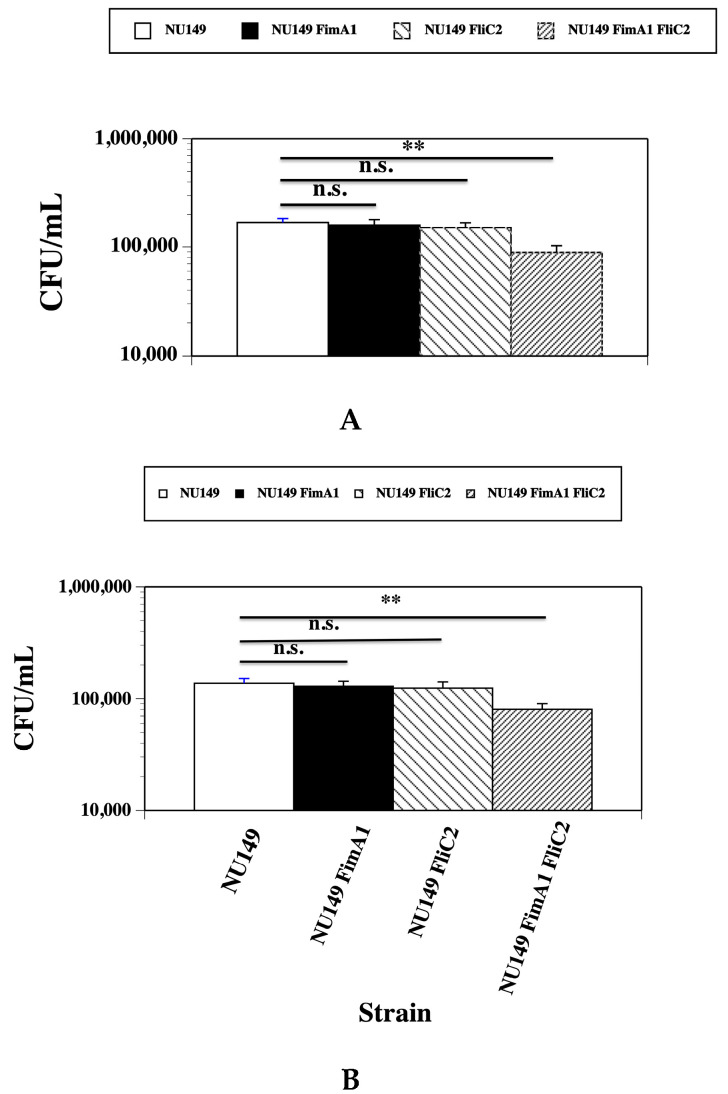
Viable count determinations of bacteria phagocytized following a one-hour exposure of UPEC strain NU149 (white column), NU149 FimA1 (black column), NU149 FliC2 (diagonal line left column), and NU149 FimA1 FliC2 (diagonal line right column) to (**A**) murine J774A.1 and (**B**) human U937 monocytes. Data is the mean ± standard deviation from three separate runs conducted in duplicate. n.s. = not significant and ** = *p* < 0.001.

From the data shown above, mutants were created that did not express type 1 pili and/or flagella. Next, the number of UPEC cells phagocytized by the monocytes was assessed. The wild type NU149, NU149 FimA1, NU149 FliC2, and NU149 FimA1 FliC2 strains were added to either the murine monocytic J774A.1 or human monocytic U937 cells and incubated for 1 h. Extracellular bacteria were killed with gentamicin, and viable counts were determined for each strain. The wild type NU149 had the highest number of phagocytized cells in both the murine J774A.1 cells (1.68 × 10^5^, Figure 2A) and the human U937 cells (1.37 × 10^5^, Figure 2B). Fewer NU149 FimA1 bacterial cells were phagocytized by J774A.1 (1.60 × 10^5^, *p* = 0.402) and U937 cells (1.28 × 10^5^, *p* = 0.322) versus the wild type cells. Phagocytosis of the NU149 FliC2 cells by J774A.1 cells (1.51 × 10^5^, *p* = 0.058) and U937 cell (1.24 × 10^5^, *p* = 0.158) was lower than either the wild type or the *fimA* mutant, but not significantly. However, the *fimA fliC* double mutant strain displayed the lowest number of cells phagocytized by J774A.1 (0.893 × 10^4^, *p* < 0.001) and U937 monocytes (8.03 × 10^4^, *p* < 0.001), which was significant when compared to the wild type cells. This data demonstrates that UPEC cells without type 1 pili and flagella were less apt to be phagocytized than the wild type UPEC cells expressing type 1 pili and flagella.

### 3.3. Loss of Type 1 Pili and Flagella Leads to Greater UPEC Survival in the Presence of Human or Murine Monocytes

**Figure 3:**
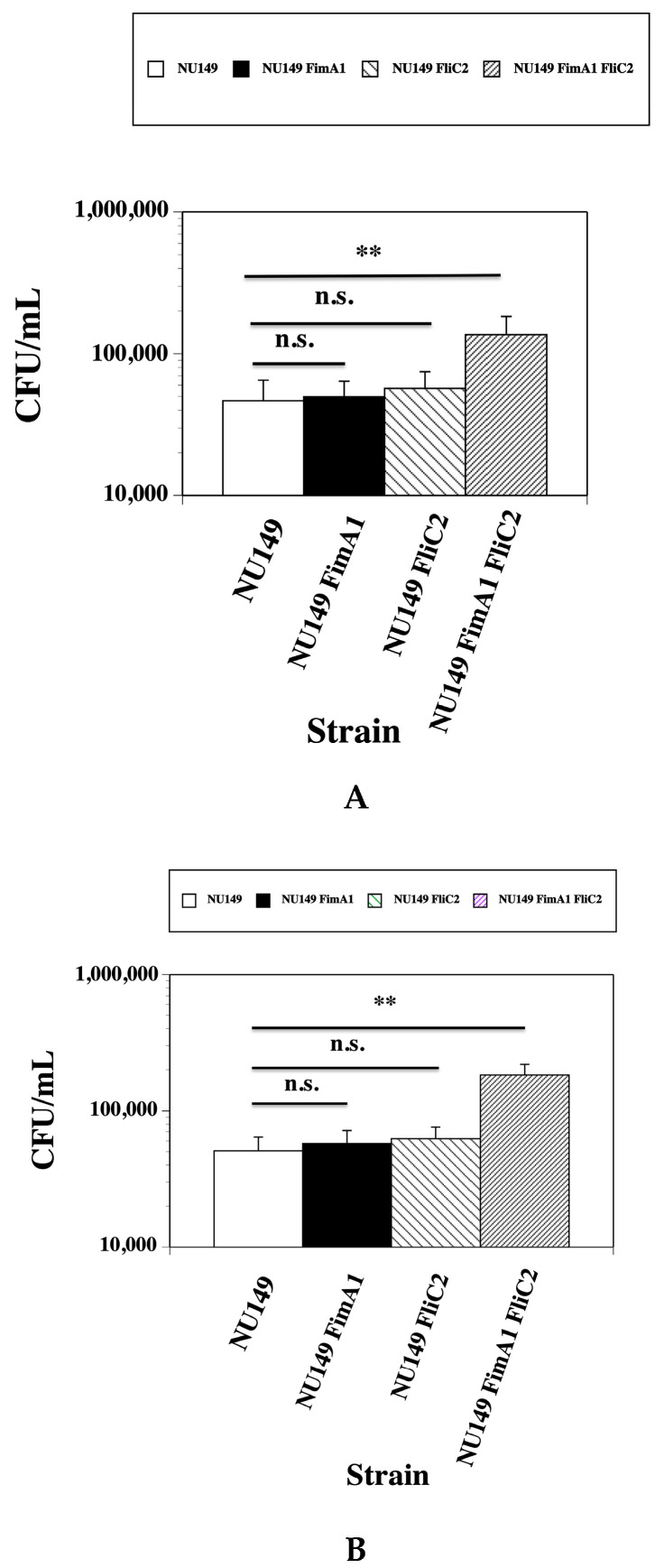
Viable count determinations of surviving bacteria following a two-hour exposure of UPEC strain NU149 (white column), NU149 FimA1 (black column), NU149 FliC2 (diagonal line left column), and NU149 FimA1 FliC2 (diagonal line right column) to (**A**) murine J774A.1 and (**B**) human U937 monocytes. Data is the mean ± standard deviation from three separate runs conducted in duplicate. n.s. = not significant and ** = *p* < 0.001.

The data above showed that the *fimA fliC* double mutant cells were phagocytized less effectively than the wild type NU149 cells by either human or murine monocytes. These mutants were tested against wild type bacteria for their ability to survive in the presence of either J774A.1 murine monocytes or U937 human monocytes. Following incubation of NU149, NU149 FimA1, NU149 FliC2, or NU149 FimA1 FliC2 with the phagocytic cells for 2 h, viable counts were performed. The wild type NU149 displayed a viable count of 4.67 × 10^4^ when added to J774A.1 cells (Figure 3A). The *fimA* mutant strain NU149 FimA1 had a slightly higher viable count of 4.98 × 10^4^, which was not significant (*p* = 0.747). A *fliC* mutant strain NU149 FliC2 had an even higher viable count of 5.7 × 10^4^, which was also not significant compared to the wild type (*p* = 0.347). However, the *fimA fliC* double mutant had a significantly higher viable count than the wild type (1.36 × 10^5^, *p* < 0.001). In the presence of murine monocytes, the double mutant survived the best.

Testing with human monocytes was then assessed. The wild type NU149 showed a viable count of 5.08 × 10^4^ (Figure 3B). Again, the *fimA* mutant strain had a slightly higher viable count of 5.73 × 10^4^ when mixed with human monocytes versus the wild type, which was not significant (0.428). The *fliC* mutant strain exhibited an even higher viable count compared to the wild type, which was also not significant (6.22 × 10^4^, *p* = 0.173). A significantly higher viable count was observed for the *fimA fliC* double mutant versus the wild type (1.83 × 10^5^, *p* < 0.001), showing that the UPEC double mutant could also survive better than the wild type in the presence of human monocytes.

## 4. Discussion

Phagocytosis is an important innate immune defense to clear bacteria from the human body [37]. In this study, the loss of type 1 pili and flagella on UPEC bacteria led to significantly less phagocytosis and killing by human and murine monocytes compared to bacteria expressing both virulence factors. Flagella are needed for ascension within the urinary tract [20,21,22,23], and type 1 pili are critical for bladder colonization and the invasion of the bladder epithelial cells [13,14,15,16,27,28], so both structures are vital for UPEC pathogenesis. However, both surface structures trigger a strong humoral immune response that can aid in the clearance of the bacteria from the human body.

Although both proteinaceous surface structures are important for UPEC cell pathogenesis, their presence triggers a release of proinflammatory cytokines. The host will elicit a strong proinflammatory cytokine response to flagella on the bacterial cells through the Toll-like receptor (TLR) 5 [38,39,40]. Type 1 pilus proteins can also elicit a strong proinflammatory cytokine response [38,41,42]. Moreover, a robust humoral immune response creates high levels of anti-flagella antibodies [43], which can lead to antibody-based opsonization and a better clearance of the bacteria.

We have shown that the UPEC strain NU149 FimA1 FliC2 mutant that was unable to express either type 1 pili or flagella was phagocytized two-fold less frequently than the wild type bacteria. Neither the Δ*fimA* mutant nor the Δ*fliC* mutant showed any significant difference between wild type and the single mutant strains in the phagocytosis assay. The presence of the other surface structure that sticks out beyond the capsule in these single mutant strains may be sufficient to facilitate the phagocytosis of the bacterial cells. No prior studies have been conducted that have examined the effects of a double *fimA fliC* mutant on UPEC uptake by phagocytic cells. Additional time points and MOIs could have led to more significant results and should be tested in the future.

Strain NU149 does not express S, P, or other pili that may be expressed by other UPEC strains like UTI89 and CFT073. Thus, in other UPEC strains, additional genes would need to be deleted. TolA [44] and OmpW [45] are other proteins tied to UPEC phagocytosis. Flagellated bacteria are more likely to be phagocytized by phagocytic cells [46]. In some environments, the loss of flagella and type 1 pili on the bacteria would allow the bacterial cells to hide behind their capsule. A capsule protects against both the phagocytosis and killing by professional phagocytic cells, like monocytes and neutrophils [47,48].

Flagella and type 1 pili are important for UPEC pathogenesis. However, the expression of both structures declines in UPEC cells colonizing the urinary tract. Over the course of a UTI, UPEC flagellin biosynthesis genes are down-regulated over the course of 10 days as the bacteria reside within murine urinary tracts [29]. UPEC type 1 pilus expression genes are also down-regulated over time in human and murine urinary tracts [26,29,33,49]. The human and murine urinary tracts are high osmolarity/acidic pH environments [50,51]. The transcription of *fliC* and several *fim* genes decreases over time when UPEC cells are bathed in an acidic/high osmolarity niche [33,49,52]. The reduction or loss of both flagella and type 1 pili in parts of these urinary tract environments could provide an advantage to some UPEC strains. Certainly, the diversity of clinical UPEC isolates, their size, and their shape can influence their engulfment by human or murine macrophages [53,54], so our results with NU149 may differ somewhat when other UPEC strains are tested.

## 5. Conclusions

Mutants of UPEC strain NU149 that had their *fimA*, *fliC*, or *fimA* and *fliC* gene(s) deleted were tested for their ability to be phagocytized by human and murine monocytes. The Δ*fimA* Δ*fliC* strain was not phagocytized as effectively and survived better than the wild type strain when mixed with human or murine monocytes, suggesting that the loss of both flagella and type 1 fimbriae provided a benefit to the bacteria in the presence of phagocytic cells.

## Figures and Tables

**Table 1:** Bacterial strains and plasmids used in this study.

Strain or Plasmid	Description	Source
Strain		
DH5α	General cloning strain	Gibco/BBL
NU149	*E. coli* cystitis isolate	[28]
NU149 FimA1	NU149 Δ*fimA*	This study
NU149 FliC2	NU149 Δ*fliC*	[24]
NU149 FimA1 FliC2	NU149 Δ*fimA* Δ*fliC*	This study
Plasmid		
pKD4	Flp recombinase sites	[32]
pKD46	λ Red recombinase	[32]
pCP20	Flp recombinase	[32]

**Table 2:** Hemagglutination titering and motility measurements for the wild type NU149 strain compared to Δ*fimA*, Δ*fliC*, and Δ*fimA* Δ*fliC* mutants.

Strain	Mutation	HA Titer ^a^	Motility ^b^
NU149	Wild type	512 ^c^	42
NU149 FimA1	Δ*fimA*	0	39
NU149 FliC2	Δ*fliC*	512	5
NU149 FimA1 FliC2	Δ*fimA* Δ*fliC*	0	3

^a^ Hemagglutination titer using 1% guinea pig erythrocytes; ^b^ Motility in soft agar measured in mm; ^c^ Mean from three runs.

## Data Availability

The raw data supporting the conclusions of this article will be made available by the authors on request.
